# Supplementary material for: A Deletion in the Canine POMC Gene Is Associated with Weight and Appetite in Obesity-Prone Labrador Retriever Dogs
Source: Cell Metab. 2016 May 10;23(5):893–900. doi: 10.1016/j.cmet.2016.04.012 (PMC4873617; doi:10.1016/j.cmet.2016.04.012)
Supplement: Document S1. Figure S1 and Tables S1–S4 [file mmc1.pdf]

Cell Metabolism, Volume *23*

Supplemental Information

A Deletion in the Canine *POMC* Gene Is Associated with Weight and Appetite in Obesity-Prone Labrador Retriever Dogs

Eleanor Raffan, Rowena J. Dennis, Conor J. O'Donovan, Julia M. Becker, Robert A. Scott, Stephen P. Smith, David J. Withers, Claire J. Wood, Elena Conci, Dylan N. Clements, Kim M. Summers, Alexander J. German, Cathryn S. Mellersh, Maja L. Arendt, Valentine P. Iyemere, Elaine Withers, Josefin Söder, Sara Wernersson, Göran Andersson, Kerstin Lindblad-Toh, Giles S.H. Yeo, and Stephen O'Rahilly

SUPPLEMENTAL INFORMATION – RAFFAN *ET AL*

SUPPLEMENTAL DATA ITEMS

Supplemental table 1 – Results of candidate gene sequencing. Related to Figure 1.

Variations (Var) from the canine reference genome (CanFam3.1) found on candidate gene sequencing. Number of dogs and their genotypes are shown. [a]V213F in MC4R has previously been shown not to affect receptor function[1]. [b]Deletion detailed in main paper. [c]rs22557623. [d]rs22557625. WT, wild type. *See Supplementalfigure 1.

Gene	Position in Ensembl CanFam3.1	Reference Allele	Mutant Allele	Consequence	Obese dogs			Lean dogs			p value of Fisher's exact test
					WT/WT	WT/Var	Var/Var	WT/WT	WT/Var	Var/Var	
MC4R	1:16132465	G	T	V213F	9	1	0	12	1	0	1.000[a]
	1:16132605	T	C	synonymous coding	0	0	9	0	0	13	Invariant
	1:16132817	G	T	S330I	9	0	0	12	1	0	1.000
	1:16132860	C	G	intronic	0	0	9	0	0	12	Invariant
	1:16133054	C	T	intronic	0	3	5	1	4	6	1.000
POMC	17:19431664	G	T	S239I	13	0	0	17	1	0	1.000
	**17:19431807**	**GCGCCGGCCCGGGA**	**-**	**P187fs**	**5**	**8**	**2**	**16**	**2**	**0**	**0.001[b]**
	17:19431813	C	G	synonymous coding	0	0	5	0	0	16	Invariant
	17:19431820	A	AG	P187PE*	0	0	15	0	0	18	Invariant
	17:19431850	C	G	R178G	0	0	12	0	0	17	Invariant
	17:19431861	A	G	K174R	0	0	12	0	0	17	Invariant
	17:19431983	C	G	synonymous coding	13	0	0	19	1	0	1.000
	17:19432208	G	A	synonymous coding	9	3	0	15	4	0	1.000
	17:19432226	C	A	synonymous coding	6	6	0	6	9	4	0.452
	17:19432391	GGGCGAGCTCCTG	-	intronic	7	4	0	10	7	0	1.000
	17:19432427	C	G	intronic	6	3	1	6	6	3	0.428[c]
	17:19432428	A	C	intronic	6	4	0	6	9	0	0.428[d]
	17:19434068	T	C	intronic	8	2	1	5	2	2	0.642

Supplemental table 2 – Breeds of dogs tested for both the *POMC* mutation and for naturally occurring variation in the *POMC* gene. Related to Figure 1.

	Number of dogs tested for:	
**Breed**	***POMC* mutation**	**Variation in *POMC* gene**
Beagle	17	2
Bearded collie	16	2
Bernese mountain dog	20	0
Border Collie	18	2
Boxer	17	2
Bull terrier	8	1
Cairn terrier	8	1
English springer spaniel	17	2
Finsk lapphund	8	1
Flat coat retriever	8	1
Giant schnauzer	16	2
Golden retriever	55	55
Great Dane	16	2
Hovawart	16	2
Irish wolfhound	16	2
Leonberger	8	1
Newfoundland	16	2
Polish lowland	8	1
Poodle	16	2
Rottweiler	20	0
Shar pei	16	2
Swedish elkhound	16	2
Welsh springer spaniel	16	2
Whippet	9	1
14 Other breeds: Crossbreed, cavalier King Charles spaniel, German shepherd, mastiff, shih tzu, Tibetan terrier, Border terrier, cocker spaniel, Dalmatian, dachshund, Doberman pinscher, pointer, Staffordshire bull terrier, West Highland white terrier	20	0

Supplemental Table 3 – Results of microsatellite genotyping in wild type and homozygous deletion Labrador retrievers and FCR. Related to figure 1.

Amplification of microsatellites 17_021D_CT (which lies 527 kb upstream of the deletion) and 17_022B_CA (66 kb downstream of the deletion) showed all FCR and Labrador retrievers homozygous for the POMC deletion were homozygous for the same microsatellite alleles, whereas wild type dogs carried various different alleles, evidence that the mutation is identical by descent in the two breeds. All map positions as per DOGSET (CanFam2)[2].

DOGSET marker name	Physical/map position on Chromosome 17 bp/centimorgans	Alleles present (length of amplicon x no. of dogs with that allele)			
		Labrador retrievers		Flat coat retrievers	
**17_021D_CT**	21885226/13.949	363/363 x 15<br>365/365 x 1	365/365 x 12	363/363 x 10<br>363/369 x 3<br>372/372 x 1	365/365 x 15
***POMC* deletion**	**22412852/13.949**	**Wild type**	**Homozygous Deletion**	**Wild type**	**Homozygous Deletion**
**17_022B_CA**	22478898/13.949	409/409 x 1<br>411/411 x 8<br>411/413 x 2<br>413/413 x 4	413/413 x 12	409/409 x 2<br>409/411 x 1<br>409/415 x 1<br>411/411 x 4<br>411/413 x 1<br>413/413 x 3	413/413 x 16
**17_022C_CA**	22558098/13.949	369/369 x 1<br>369/371 x 3<br>369/375 x 2<br>371/371 x 2<br>371/375 x 2<br>375/375 x 4	371/371 x 10<br>369/369 x 1<br>371/373 x 1	369/369 x 6<br>369/371 x 4<br>371/371 x 1<br>371/375 x 2	371/371 x 9<br>371/369 x 3<br>369/369 x 1

Supplemental Table 4 – Distribution of alleles in working and show strains of Labrador retrievers

There was no difference between the incidence of the mutation between dogs from 'working' and 'show' lines (Chi-squared test of wild type vs. carrier $p>0.3$). Related to figure 1.

	Wild Type	Heterozygous	Homozygous Deletion
**Working**	94	27	3
**Show**	21	9	1

Supplemental Figure 1 – Clustal Omega alignment of the CanFam3.1 reference sequence with sequence in Labrador retrievers and other breeds for exons 3-4 of POMC. Related to Figure 1. Alignment annotated to show variation from the reference sequence identified in Labrador retrievers and other breeds (see Supplementaltable 1). Underlined bases denote position of frameshift deletion. (a) 17: 19431861, A>G in all dogs sequenced with consequence p.K174R, which changes the distal di-basic cleavage site for ACTH to 'KR' from 'KK' which is the same as the human reference sequence. (b) 17: 19431850 C>G in all dogs sequenced, consequence p.R178G which is identical to the human reference sequence. (c) 17:19431821 C>CG. This single base pair insertion at the start of what is annotated as a two base pair intron (highlighted blue) has the consequence p.P187>PE which results in closer homology to the human reference sequence. (d) 17:19431813 C>G in all dogs sequenced, synonymous coding. (e) 17:19431777 C>T, novel synonymous coding SNP. (f) 17:19431764 G>T, p.E206D, not found in Labrador retrievers or FCR.

```
CLUSTAL O(1.2.1) multiple sequence alignment

POMC_reference_CanFam3.1      GCGTGCATCCGGGCCTGCAAGCCCGACCTCTCCGCCGAGACGCCCGTGCTCCCCGGCAAC
All_breed_consensus           GCGTGCATCCGGGCCTGCAAGCCCGACCTCTCCGCCGAGACGCCCGTGCTCCCCGGCAAC
                              ************************************************************

POMC_reference_CanFam3.1      GGCGACGAGCAGCCGCTGGCTGAGAACCCCCGGAAGTACGTCATGGGCCACTTCCGCTGG
All_breed_consensus           GGCGACGAGCAGCCGCTGGCTGAGAACCCCCGGAAGTACGTCATGGGCCACTTCCGCTGG
                              ************************************************************

POMC_reference_CanFam3.1      GACCGGTTTGGCCGCCGCAATGGCAGCGCGGGCCAGAAGCGCGAGGAAGAAGAGGTGGCG
All_breed_consensus           GACCGGTTTGGCCGCCGCAATGGCAGCGCGGGCCAGAAGCGCGAGGAAGAAGAGGTGGCG
                              ************************************************************

POMC_reference_CanFam3.1      GCGGGCGGAGGCCGCGCCCCGCTGCCCGCGGGCGGCCCGGGGCCCCGCGGCGACGGTGGC
All_breed_consensus           GCGGGCGGAGGCCGCGCCCCGCTGCCCGCGGGCGGCCCGGGGCCCCGCGGCGACGGTGGC
                              ************************************************************

POMC_reference_CanFam3.1      GAGCTCGGCCTGCAAGAGGGCAAGCGCTCCTACTCCATGGAGCACTTCCGCTGGGGCAAG
All_breed_consensus           GAGCTCGGCCTGCAAGAGGGCAAGCGCTCCTACTCCATGGAGCACTTCCGCTGGGGCAAG
                              ************************************************************

POMC_reference_CanFam3.1      CCGGTGGGCAAGAAGCGGCGCCCGGTGAAGGTGTACCCCAACGGCGCTGAGGACGAGTCG
All_breed_consensus           CCGGTGGGCAAGAAGCGGCGCCCGGTGAAGGTGTACCCCAACGGCGCTGAGGACGAGTCG
                              ************************************************************

POMC_reference_CanFam3.1      GCCGAGGCCTTCCCCGTCGAGTTCAAGAAGGAGCTGGCCCGGCAGCGGCTGGAGCCGGCG
All_breed_consensus           GCCGAGGCCTTCCCCGTCGAGTTCAAGAGGGAGCTGGCCGGGCAGCGGCTGGAGCCGGCG
                              **************************** ********** ********************
                                                         (a)        (b)
POMC_reference_CanFam3.1      CTCGGCCCC-AGGGCCCCGCCGCGGGCGTGGCGGCGCTGGCCGACCTGGAGTACGGCCTG
All_breed_consensus           CTCGGCCCCGAGGGCCCGGCCGCGGGCGTGGCGGCGCTGGCCGACCTGGAGTAYGGCCTG
                              ********* ******* *********************************** ******
                                      (c)     (d)                                 (e)
POMC_reference_CanFam3.1      GTGGCGGAGGCCGGGGCGGCCGAGAAGAAGGACGACGGGCCCTACAAGATGGAGCACTTC
All_breed_consensus           GTGGCGKAGGCCGGGGCGGCCGAGAAGAAGGACGACGGGCCCTACAAGATGGAGCACTTC
                              ****** *****************************************************
                                   (f)
POMC_reference_CanFam3.1      CGCTGGGGCAGCCCGCCCAAGGACAAGCGCTACGGCGGCTTCATGAGCTCGGAGAGGAGC
All_breed_consensus           CGCTGGGGCAGCCCGCCCAAGGACAAGCGCTACGGCGGCTTCATGAGCTCGGAGAGGAGC
                              ************************************************************

POMC_reference_CanFam3.1      CAGACGCCCCTGGTGACGCTGTTCAAAAACGCCATCATCAAGAACGCCCACAAGAAGGGC
All_breed_consensus           CAGACGCCCCTGGTGACGCTGTTCAAAAACGCCATCATCAAGAACGCCCACAAGAAGGGC
                              ************************************************************

POMC_reference_CanFam3.1      CAGTGA
All_breed_consensus           CAGTGA
                              ******
```

Supplemental References

1 Yan, J. & Tao, Y. X. Pharmacological characterization of canine melancortin-4 receptor and its natural variant V213F. *Domestic animal endocrinology* **41**, 91-97, doi:10.1016/j.domaniend.2011.05.002 (2011).

2 Wong, A. K. & Neff, M. W. DOGSET: pre-designed primer sets for fine-scale mapping and DNA sequence interrogation in the dog. *Animal genetics* **40**, 569-571, doi:10.1111/j.1365-2052.2009.01875.x (2009).
