# Supplementary material for: A Deletion in the Canine POMC Gene Is Associated with Weight and Appetite in Obesity-Prone Labrador Retriever Dogs
Source: Cell Metab. 2016 May 10;23(5):893–900. doi: 10.1016/j.cmet.2016.04.012 (PMC4873617; doi:10.1016/j.cmet.2016.04.012)
Supplement: Document S2. Article plus Supplemental Information [file mmc2.pdf]

Short Article

Cell Metabolism

A Deletion in the Canine *POMC* Gene Is Associated with Weight and Appetite in Obesity-Prone Labrador Retriever Dogs

Graphical Abstract

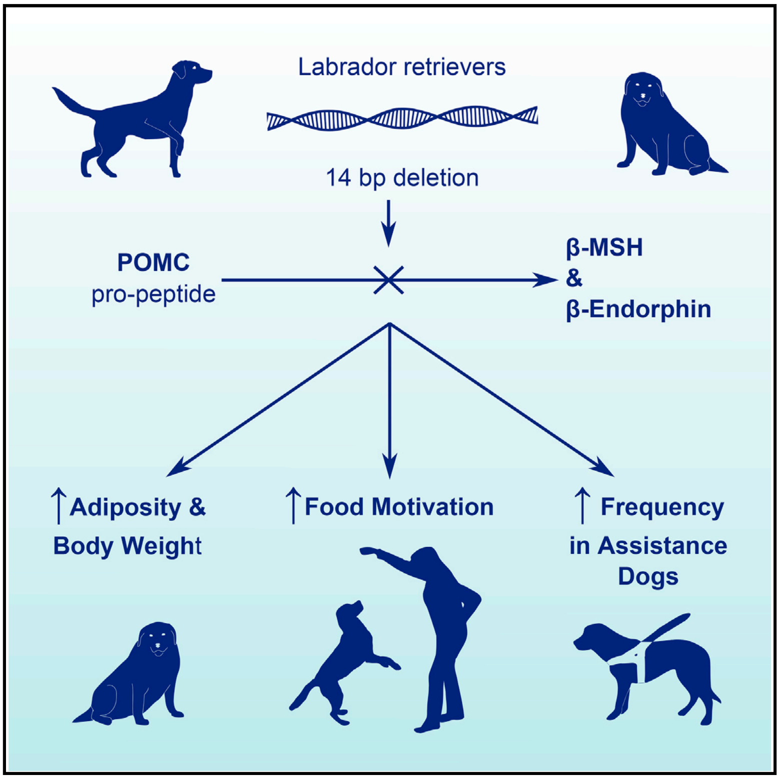

Highlights

- A *POMC* mutation is common in the obesity-prone Labrador retriever breed of dog
- It disrupts β-MSH and β-endorphin production, both implicated in energy homeostasis
- Mutation is absent from other breeds except related flat-coat retrievers
- The mutation is associated with weight, adiposity, and food motivation in both breeds

Authors

Eleanor Raffan, Rowena J. Dennis, Conor J. O'Donovan, ..., Kerstin Lindblad-Toh, Giles S.H. Yeo, Stephen O'Rahilly

Correspondence

er311@cam.ac.uk (E.R.),
so104@medschl.cam.ac.uk (S.O.)

In Brief

Companion dogs from the obesity-prone Labrador retriever breed were found to carry a mutation in *POMC* in this study by Raffan et al. The mutation is predicted to disrupt production of the neuroactive peptides β-MSH and β-endorphin and was associated with greater weight, adiposity, and food motivation in affected dogs.

Raffan et al., 2016, Cell Metabolism *23*, 893–900
May 10, 2016 © 2016 The Authors. Published by Elsevier Inc.
http://dx.doi.org/10.1016/j.cmet.2016.04.012

CellPress

A Deletion in the Canine *POMC* Gene Is Associated with Weight and Appetite in Obesity-Prone Labrador Retriever Dogs

Eleanor Raffan,[1,14,*] Rowena J. Dennis,[1] Conor J. O'Donovan,[1] Julia M. Becker,[1] Robert A. Scott,[2] Stephen P. Smith,[3] David J. Withers,[1] Claire J. Wood,[1] Elena Conci,[1] Dylan N. Clements,[4,5] Kim M. Summers,[4] Alexander J. German,[6] Cathryn S. Mellersh,[7] Maja L. Arendt,[8] Valentine P. Iyemere,[1] Elaine Withers,[1] Josefin Söder,[9] Sara Wernersson,[9] Göran Andersson,[10] Kerstin Lindblad-Toh,[11,12] Giles S.H. Yeo,[1,13] and Stephen O'Rahilly[1,13,*]
[1]University of Cambridge Metabolic Research Laboratories
[2]MRC Epidemiology Unit
WT-MRC Institute of Metabolic Science, University of Cambridge, Cambridge CB2 0QQ, UK
[3]School of Clinical Medicine, University of Cambridge, Cambridge CB2 0SP, UK
[4]The Roslin Institute
[5]The Royal (Dick) School of Veterinary Studies
University of Edinburgh, Easter Bush, Midlothian EH25 9RG, UK
[6]Institute of Ageing and Chronic Disease, University of Liverpool, Neston, Cheshire CH64 7TE, UK
[7]Department of Canine Genetics, Animal Health Trust, Newmarket, Suffolk CB8 7UU, UK
[8]IMBIM, Uppsala University, Uppsala 75123, Sweden
[9]Department of Anatomy, Physiology, and Biochemistry
[10]Department of Animal Breeding and Genetics
Swedish University of Agricultural Sciences, Uppsala 75007, Sweden
[11]Broad Institute of Harvard and MIT, Cambridge, MA 02142, USA
[12]Science for Life Laboratory, Uppsala 75123, Sweden
[13]Co-senior author
[14]Twitter: @GOdogsProject
*Correspondence: er311@cam.ac.uk (E.R.), so104@medschl.cam.ac.uk (S.O.)
http://dx.doi.org/10.1016/j.cmet.2016.04.012

SUMMARY

Sequencing of candidate genes for obesity in Labrador retriever dogs identified a 14 bp deletion in *pro-opiomelanocortin* (*POMC*) with an allele frequency of 12%. The deletion disrupts the β-MSH and β-endorphin coding sequences and is associated with body weight (per allele effect of 0.33 SD), adiposity, and greater food motivation. Among other dog breeds, the deletion was only found in the closely related flat-coat retriever (FCR), where it is similarly associated with body weight and food motivation. The mutation is significantly more common in Labrador retrievers selected to become assistance dogs than pets. In conclusion, the deletion in *POMC* is a significant modifier of weight and appetite in Labrador retrievers and FCRs and may influence other behavioral traits.

INTRODUCTION

In developed countries, the prevalence of canine obesity ranges between 34% and 59% (Colliard et al., 2006; Courcier et al., 2010; Edney and Smith, 1986; Lund et al., 2006; McGreevy et al., 2005). Obesity in dogs is associated with reduced lifespan and several specific morbidities similar to those seen in human obesity (German, 2006; Lawler et al., 2008; Raffan, 2013; Zoran, 2010). Recent changes in the prevalence of obesity in dogs mirror increases in the prevalence of the human condition, and similar environmental factors such as reduced exercise and ready access to high-calorie food are implicated. However, despite the fact that dog owners control their pets' diet and exercise, susceptibility to obesity varies between dog breeds, which suggests the influence of genetic factors.

Over the past 20 years, insights from human and mouse genetics have illuminated multiple pathways within the brain that play a key role in the control of food intake (Yeo and Heisler, 2012). In particular, we now know that the hypothalamic leptin melanocortin signaling pathway is crucial for the appropriate control of food intake, with genetic disruption of most components of the pathway resulting in severe obesity in both mouse and man (Yeo and Heisler, 2012). However, the majority of common obesity in humans is polygenic, with the most reproducible finding from genome-wide association studies, an association at the fat mass and obesity (*FTO*) locus, explaining only a small component of obesity risk (Tung et al., 2014).

Of all dog breeds for which data have been reported, Labrador retrievers have the greatest documented obesity prevalence (Edney and Smith, 1986; Lund et al., 2006; Mason, 1970; O Neill et al., 2014) and have been shown to be more food motivated than other breeds (Raffan et al., 2015). The fact that most modern dog breeds originated relatively recently from a small number of founder animals makes the genetic

This is an open access article under the CC BY license (http://creativecommons.org/licenses/by/4.0/).

A

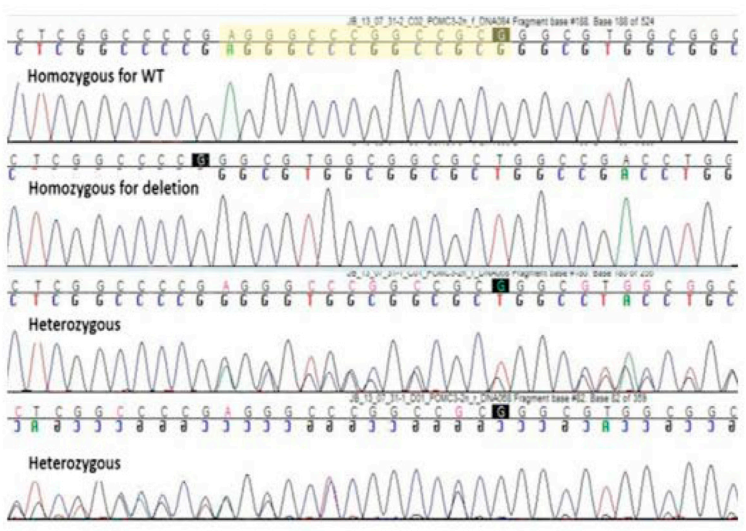

B

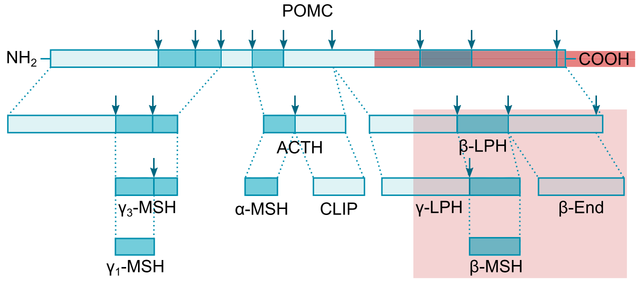

C

```
POMC-human       MPRSCCSRSGALLLALLLQASMEVRGWCLESSQCQDLTTESNLLECIRAC 50
POMC-Labrador    MPRSCCSRPGALLLALLLQASVEVSGWCLESSQCQDLTTESNLLACIRAC 50
                 ********.************:** ******************** *****
POMC-deletion    identical to Labrador wild type                    50

POMC-human       KPDLSAETPMFPGNGDEQPLTENPRKYVMGHFRWDRFGRRNSSSSGSSGA 100
POMC-Labrador    KPDLSAETPVLPGNGDEQPLAENPRKYVMGHFRWDRFGRRN----GSAGQ 96
                 *********::*********:*********************    **:*
POMC-deletion    identical to Labrador wild type                    96

POMC-human       GQKREDVSAGEDCGPLPEGGPEPRSDGAKPGPREGKRSYSMEHFRWGKPV 150
POMC-Labrador    KREEEEVAAGGGRAPLPAGGPGPRGDGGELGLQEGKRSYSMEHFRWGKPV 146
                 ::.*:*:** . .*** *** **.**.: * :******************
POMC-deletion    identical to Labrador wild type                    146

POMC-human       GKKRRPVKVYPNGAEDESAEAFPLEFKRELTGQRLREGDGPDGPADDGAG 200
POMC-Labrador    GKKRRPVKVYPNGAEDESAEAFPVEFKRELARQRLEPALGPEGPAAG-VA 195
                 ***********************:***:**: ***. . **:*** . ..
POMC-deletion    identical to Labrador wild type----------GRGGAGRPG 196

POMC-human       AQADLEHSLLVAA---EKKDEGPYRMEHFRWGSPPKDKRYGGFMTSEKSQ 247
POMC-Labrador    ALADLEYGLVAEAGAAEKKDDGPYKMEHFRWGSPPKDKRYGGFMSSERSQ 245
                 * ****:.*:. *   ****:***:******************:**:**
POMC-deletion    VRPGGGGRGGREEGRRALQDGALPLGQPAQGQALRRLHELGEEPDAPGDA 246

POMC-human       TPLVTLFKNAIIKNAYKKGE 267
POMC-Labrador    TPLVTLFKNAIIKNAHKKGQ 265
                 ***************:***:
POMC-deletion    VQKRHHQERPQEGPVRAQRGRRLLFPGSPRKAPLLPALLPSRPPPAPDVL 296

POMC-deletion    LAQAVGGSPVVSLLKLPVVRK 317
```

(legend on next page)

basis of canine traits particularly amenable to dissection (Sutter and Ostrander, 2004).

In order to begin exploiting the power of canine genetics to identify alleles predisposing to obesity, we studied a cohort of companion and assistance Labrador retriever dogs. Here, we report that a 14 bp deletion in the *pro-opiomelanocortin* (*POMC*) gene, which results in the disruption of β-MSH (melanocyte-stimulating hormone) and β-endorphin, is associated with increased body weight, adiposity, and food motivation in both Labrador retrievers and the closely related flat-coat retrievers (FCRs). We also find that the mutation is significantly more common in Labradors selected to become assistance dog breeding stock than those selected to be companions.

RESULTS

Identification of a *POMC* Deletion in Obese Labrador Retrievers

We recruited a cohort of 310 pet and assistance dog Labrador retrievers. Dogs were weighed, and their body condition score (a validated measure of adiposity that accounts for variation in body morphology within and across breeds; Laflamme, 1997; Mawby et al., 2004) was assessed by independent veterinary professionals.

Initially, the coding sequence of three candidate obesity genes, melanocortin-4 receptor (*MC4R*), agouti-related peptide (*AGRP*), and *POMC*, all part of the hypothalamic melanocortin pathway, was examined in 15 obese and 18 lean Labrador retrievers. No variants from the reference sequence were identified in *AGRP*. In *MC4R*, four novel variants were identified, but there was no significant difference in distribution of the variants between lean and obese groups (Table S1, available online).

In *POMC*, 11 novel variants were identified (Table S1), but only one was distributed differently between lean and obese groups: a 14 bp deletion at position 17:19431807-19431821 was found in 10/15 obese dogs (two homozygous and eight heterozygous) and 2/18 lean dogs (both heterozygous). The deletion spanned what is annotated in the canine reference sequence (CanFam3.1) as a 2 bp intron between exons 3 and 4. However, all Labrador retrievers and 35 dogs of 24 other breeds in which the region was re-sequenced (Table S2) were found to have the same single base pair insertion at 17:19431820 (T/TC), which has the effect of adding a base to the ''intron'' and encoding an extra amino acid (POMC p.P187 > PE). Sequence alignment shows this increases similarity to the human reference sequence (Figures 1 and S1). A TaqMan assay was developed for subsequent genotyping of the mutation.

Association of *POMC* Deletion with Weight, Adiposity, and Food Motivation in Labrador Retrievers

The association of the deletion with body weight and adiposity was tested in the wider cohort of 310 Labrador retrievers. The POMC deletion was positively associated with higher body weight ($p < 0.0001$; mean effect size 1.90 kg per deletion allele, equivalent to 0.33 SDs) and body condition score ($p < 0.0001$; mean effect size 0.48 scale point increase per deletion allele) (Figure 2). Further, food motivation, tested using the previously validated Dog Obesity Risk Assessment (DORA) questionnaire (Raffan et al., 2015), was positively associated with the presence of the mutation ($p = 0.001$; mean effect size 9.9% per deletion allele) (Figure 2).

The POMC Deletion Is Found in FCRs, Where It Is Also Associated with Weight and Food Motivation

The mutation was absent from dogs of 38 other diverse breeds (Table S2) but present in FCRs. In a sample of 96 unrelated FCRs, the allelic frequency of the *POMC* deletion was 60%, with genotypes distributed approximately evenly (32% wild-type, 29% heterozygous, and 39% homozygous deletion), indicating significant divergence from Hardy-Weinberg equilibrium ($p = 0.01$). FCRs are closely related to Labrador retrievers (Vonholdt et al., 2010); both breeds originated in the 19th century from a now extinct breed, the St. John's water dog. In both breeds, the mutation was associated with identical alleles at flanking microsatellite markers 600 kb apart, indicating identity by descent and a common ancestral origin of the mutation (Table S3).

We recruited 200 further FCRs for genotyping and phenotypic assessment. The *POMC* mutant allele was associated with higher body weight ($p < 0.0001$; mean effect size 1.86 kg per deletion allele, equivalent to 0.33 SDs) and food motivation ($p < 0.0001$; mean effect size 7.4% per deletion allele) (Figure 3).

Canine β-MSH Is Produced and Activates Melanocortin Receptors

POMC is translated as a pro-protein, and a series of bioactive peptides are produced by proteolytic cleavage. The deletion (POMC p.E188fs) is predicted to disrupt the coding sequence of *POMC* and cause loss of production of β-MSH and β-endorphin. β-MSH is a known product of the human *POMC* gene, but in the rodent genome, the N-terminal proteolytic processing site that precedes β-MSH is absent. Canine POMC has greater similarity to the human peptide sequence (79%) than does the mouse (69%). All proteolytic cleavage sites critical to processing human POMC, including those flanking β-MSH, are conserved in the dog, and the peptide sequence of the produced cleavage products is either identical or very similar (Figure 1C).

Figure 1. A 14 bp Deletion in Canine *POMC* at Position 17: 19431807-19431820 Causes a Frameshift Mutation in the Coding Sequence, Predicted to Stop Production of the Neuroactive Peptide Derivatives β-MSH and β-Endorphin

(A) Chromatogram showing capillary sequencing results from wild-type, homozygous deletion, and heterozygous dogs.

(B) Schematic diagram of POMC showing the pro-peptide and cleavage products. Arrows show di-basic cleavage sites. The mutant peptide is indicated by the position of the red line and results in loss of sequence homology from amino acid 187 of the wild-type dog POMC and a pro-peptide that is 52 amino acids longer. The red box indicates the downstream products that are not produced as a consequence of the mutation.

(C) Alignment of human and canine wild-type POMC and the peptide sequence resulting from the frameshift mutation. Sequence similarity is high (79%) between human and wild-type dog sequences, particularly for cleavage sites (bold type) and neuroactive peptides (highlighted in color). Blue: γ-MSH (human 138–150), ACTH (human 138–176), and α-MSH (human 138–150). Red, peptide products disrupted by mutation: β-MSH (human 217–234) and β-endorphin (human 237–267).

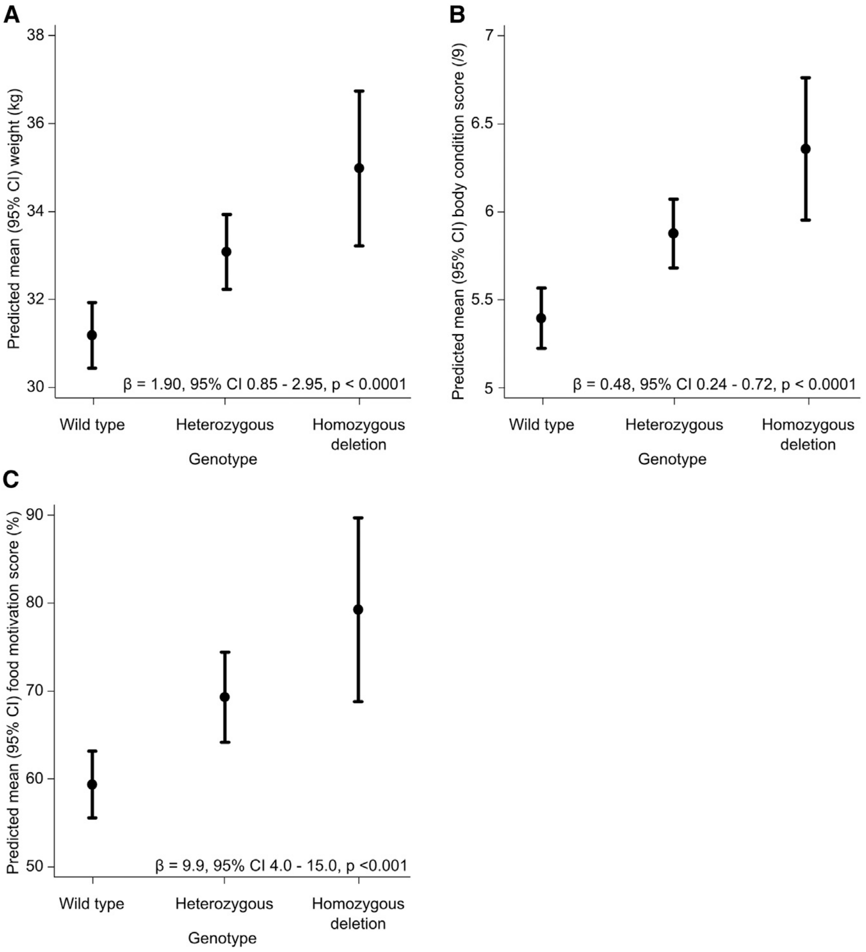

Figure 2. Effect of POMC Deletion Genotype on Measures of Obesity and Food Motivation in Labrador Retrievers

Predicted mean (95% confidence interval) (A) weight (n = 258), (B) body condition score (n = 236), and (C) food motivation score (n = 210) by genotype after linear regression adjustment for age, sex, neuter status, and color are shown. Mean effect size (β), 95% confidence interval, and p values are shown.

The MSH peptides exert their actions on body weight through two closely related G protein-coupled melanocortin receptors. To test whether canine β-MSH and α-MSH have comparable receptor-activating effects on their cognate receptors, we cloned the canine *MC3R* and *MC4R* and expressed them in Cos-7 cells, before treating with α-MSH or β-MSH (both canine and human) and testing the downstream response by measuring cAMP (cyclic AMP) concentrations. The activity profiles of canine α- and β-MSH on the canine MC3R and MC4R were indistinguishable from those of the human ligands on the human receptors (Figure 4).

Frequency of POMC Deletion Is Higher in Assistance Dog Populations

In studies designed to more robustly determine the prevalence of this deletion among Labrador retrievers, we accessed DNA from further cohorts of dogs. Of 383 Labrador retrievers from the UK, 78% of dogs were wild-type, 20% heterozygous, and 2% homozygous deletion (allele frequency 12%, proportions in Hardy-Weinberg equilibrium, statistically similar to the population used to test association with obesity and food motivation); allele frequencies were the same in 28 Labrador retrievers from the United States. Notably, in a group of 81 Labrador retrievers used as assistance dog breeding stock, the allelic frequency was markedly higher at 45%. It is also of note that the allelic distribution in the assistance dogs was markedly out of Hardy-Weinberg equilibrium (23% wild-type, 64% heterozygous, and 12% homozygous deletion; $p < 0.01$), suggesting the possibility of positive selection toward heterozygous dogs in that population.

DISCUSSION

We have shown that a frameshift deletion mutation in *POMC* is strongly associated with weight, adiposity, and appetite in Labrador retriever and FCR dogs. In both breeds, each deletion allele confers an increase of 0.33 SDs in weight. This effect size (over three times the per-allele effect observed at the *FTO* locus in humans; Locke et al., 2015) is particularly notable given the extent to which owners, rather than the dogs themselves, control the amount of food and exercise dogs receive.

While a previous study reported two non-coding SNPs in *tumor necrosis factor (TNF)* were associated with increased body condition score in Labradors, the cohort size was small, and it did not address the crucial phenotypes of weight or food motivation (Mankowska et al., 2016). It has been reported that owners of more highly food-motivated dogs make greater efforts to limit their dogs' access to food (Raffan et al., 2015). However, there is evidence to suggest dogs are able to influence both the type and quantity of food offered to them by their owners (Day et al., 2009). It is possible that behavior changes related to the mutation are sufficient to lead to increased food intake (either by scavenging or soliciting owner-provided food). Furthermore, it is well recognized that in mice, failure of signaling at the MC4R enhances caloric efficiency (Ste Marie et al., 2000), a phenotype we cannot exclude here.

The association of the *POMC* deletion described with body weight, adiposity, and food motivation in both Labrador retrievers and FCRs is strong and of a similar magnitude in both breeds. It is possible that the high prevalence of the mutation in Labrador retrievers contributes to their well-known predisposition to obesity compared to other breeds of dog. We acknowledge that there is a higher prevalence of this deletion in FCRs, a breed not previously noted to be especially obesity prone, but note that obesity has not been studied systematically before in this relatively uncommon breed of dog.

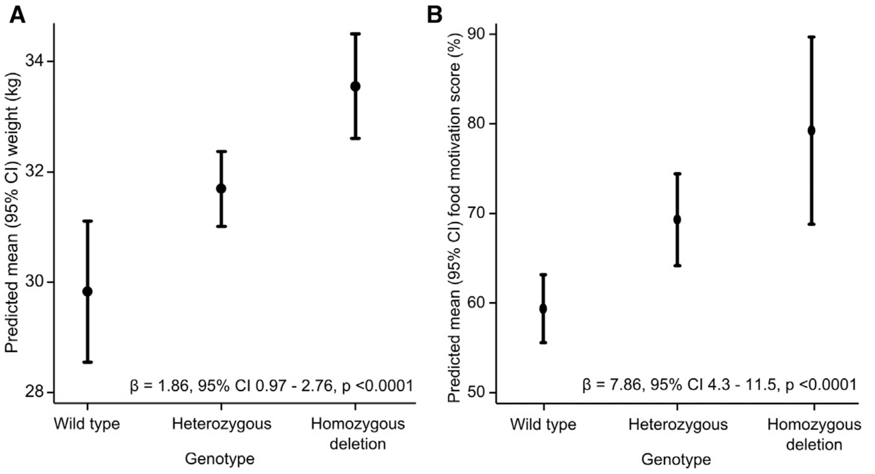

Figure 3. Effect of POMC Deletion Genotype on Weight and Food Motivation in FCRs

Predicted mean (95% confidence interval) (A) weight (n = 196) and (B) food motivation score (n = 219) by genotype after linear regression adjustment for age, sex, neuter status, and color are shown. Mean effect size β, 95% confidence interval and p values are shown.

The particularly high allelic frequency of the *POMC* deletion in assistance dogs is intriguing. Temperament and "trainability" are the main drivers for selection of assistance dogs, and "positive reinforcement" with food reward is a mainstay of puppy training. We therefore hypothesize that dogs carrying the *POMC* deletion may be more likely to be selected as assistance dogs. The fact that the allelic frequency at this locus is significantly out of Hardy-Weinberg equilibrium in assistance dog breeding stock could be seen as support for the notion that selection has occurred at this locus.

The mechanism by which reduced β-MSH and β-endorphin due to the mutation causes behavioral and weight phenotypes remains to be precisely elucidated. Previously, study of the role of β-MSH in regulation of appetite and energy homeostasis has been limited by the fact that rodents lack the proximal di-basic cleavage site necessary for the proteolytic cleavage event that produces β-MSH, and the scarcity of human patients lacking functional β-MSH (Challis et al., 2002; Lee et al., 2006). However, studies of humans with POMC mutations resulting in aberrant forms of β-MSH (due to disruption of the receptor binding site; Lee et al., 2006; or production of an aberrant β-MSH/β-endorphin fusion protein; Challis et al., 2002) have suggested that β-MSH is important in controlling appetite and obesity development in man, with hyperphagia notable in patients with both mutations. Our current observation that the relationship between weight, body condition score, and appetite and genotype is proportional to the number of mutant alleles present is consistent with the fact that these human β-MSH mutations were found in heterozygous form (Challis et al., 2002; Lee et al., 2006). This is likely due, as in humans, to straightforward haploinsufficiency, but alternative explanations (e.g., altered splicing leading to production of an aberrant peptide acting as an antagonist at the MC4R) exist. Ideally, the sequence and relative expression of *POMC* RNA transcripts would be examined, but the difficulty of obtaining suitable samples from a companion dog cohort precludes this at present.

The role of β-endorphin in regulating appetite, satiety, and energy balance is less well understood, but it has been proposed to underlie oro-sensory reward in high-need states or when the stimulus is especially valuable (Mendez et al., 2015). However, mice selectively lacking β-endorphin are hyperphagic and obese, suggesting that the loss of both neuropeptides could contribute, in combination, to the phenotype seen in dogs carrying this frameshift *POMC* mutation (Appleyard et al., 2003).

In conclusion, further study of dogs naturally lacking these important bioactive peptides encoded by the *POMC* gene should provide novel information regarding melanocortin and opioid biology and opens up potential novel therapeutic approaches to at least some forms of canine obesity.

EXPERIMENTAL PROCEDURES

Ethical Approval

The research was approved by the Ethical Review Committee of the Department of Veterinary Medicine, University of Cambridge (CR73 and CR125), with sample collection at other centers also approved by local ethical review committees: Animal Health Trust Research Ethics Committee, MIT Animal Care Protocol Lindblad-Toh 0913-073-16, Veterinary Ethical Review Committee of the University of Edinburgh (VERC 11/12), University of Liverpool Research Ethics Committee RETH000353, Swedish Animal Ethical Committee (C138/12, C62/10, and C2/12), and the Swedish Animal Welfare Agency (no. 31-1711/10). All dog owners gave full written consent to participate in the research.

Dogs to Test Genotype/Obesity Association

Labrador retriever samples were collected from dogs from a large assistance dog breeding colony (n = 81) or that were pet dogs from the UK (n = 310). Pet dogs were recruited either after their owners volunteered in response to an email from the UK Kennel Club sent to over 15,000 Labrador retriever owners, or via participating veterinary practices. Inclusion and exclusion criteria were set in advance of dog recruitment. Inclusion criteria were that dogs must be greater than 1 year of age and have submitted DNA and at least one of the following: weight, body condition score, or DORA questionnaire response, along with data regarding age, gender, and color. Exclusion criteria were current treatment with drugs that could affect appetite or weight (e.g., corticosteroids or anti-seizure medication) or being under veterinary care for diagnosis or treatment of ill health (12 dogs excluded).

Labrador retrievers included in the analysis had mean (SD) age of 6.1 years (2.6), weight of 32 kg (5.7), body condition score of 5.6 units (1.2), and food motivation score of 63% (25). Half were male and half female; 56% of both sexes were neutered. Fifty-six percent of dogs were black, 34% yellow, and 10% chocolate.

FCRs were recruited via a Kennel Club email request for volunteers sent to over 7,000 owners of FCRs. DORA questionnaire responses, body weight, and DNA samples were collected from all participant dogs, but body condition scores were not available. Inclusion criteria were availability of DNA, DORA questionnaire response, and weight, along with data regarding age, gender, and color. FCRs were excluded from the analysis if they were less than 1 year old or owners reported specific ill health. Health information was gathered by review of veterinary clinical records by a veterinary surgeon (ER).

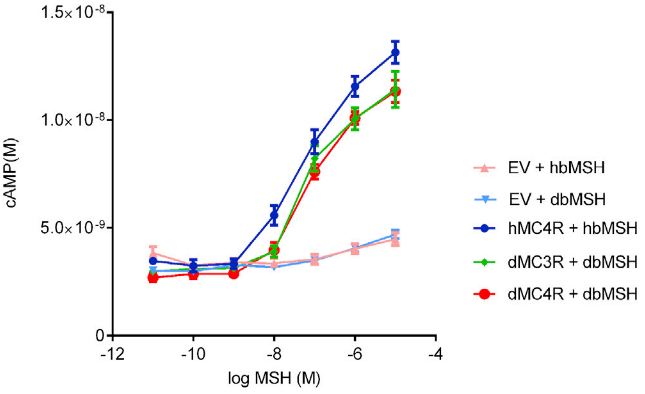

Figure 4. Activity of Canine β-MSH on MC4R and MC3R

Canine β-MSH acts on the canine melanocortin receptors in a similar fashion to the analogous human receptor/ligand pairs. Equivalent results were obtained when human and dog MC4R and MC3R were stimulated with α-MSH (Error bars represent SEM).

FCRs included in the analysis had mean (SD) age of 5.3 years (2.0), weight of 32 kg (5.6), and food motivation score of 67% (22). Fifty-two percent were male (55% neutered) and 48% female (68% neutered). Eighty-five percent of FCRs were black and 15% liver in color.

Assessment of Weight, Adiposity, and Food Motivation

All dogs were weighed on electronic scales. Body condition scores were assessed by qualified veterinary professionals who used a previously validated standard chart (Laflamme, 1997; Mawby et al., 2004). Food motivation was assessed by use of the previously validated DORA questionnaire, which is an owner-reported measure of dogs' behavior related to food, as tested using a panel of questions (Raffan et al., 2015).

Dogs Used to Test Population Distributions

The population distribution of the *POMC* mutation was tested in each affected breed in cohorts of dogs collected without reference to obesity or weight; 383 British Labrador retrievers, 28 American Labrador retrievers, and 96 FCRs were screened. Other breed samples were from unrelated dogs from the United States or Sweden.

Genomic DNA Sequencing

DNA was extracted from saliva samples collected using Performagene (PG-100) kits (DNA Genotek). PCR primers were designed to amplify coding regions and flanking intronic regions of candidate genes. Initial sequencing was performed in 18 lean Labrador retrievers, selected because their owners reported the dogs were not very food motivated, and 15 obese Labrador retrievers. Sequences were aligned using Sequencher software (Gene Codes) against the canine reference sequence (CanFam3.1).

Genotyping the POMC Deletion

A TaqMan genotyping assay was developed to allow rapid genotyping of the mutation. A single pair of primers flanking the deletion site and paired probes that detected either the wild-type or mutant allele sequence were combined in a duplex reaction. After optimization of reaction efficiency and detection thresholds, Ct (cycle threshold) values within 0.5 cycles were expected for heterozygous samples. Where Ct > 10 for both alleles but delta Ct was >0.5, the same primer pair was used to amplify the fragment by standard PCR with subsequent size separation using electrophoresis on a 3% TBE/agarose gel and visualization of wild-type, deletion, or both alleles against reference controls. Primer sequences are available upon request. Investigators performing genotyping were blinded to the phenotype of the dogs.

MC4R and MC3R Cloning

The coding sequence of the single exons of *MC4R* and *MC3R* were amplified from dog (Labrador retriever) genomic DNA using *Pfu* Ultra Fusion HS DNA Polymerase (Agilent Technologies) according to the manufacturer's instructions. Restriction sites for *EcoR1* and *Xba1* were introduced into the amplicon by incorporation in the PCR primers to allow subsequent sub-cloning into the pFLAG-CMV-2 vector (Sigma-Aldrich) with restriction enzymes *EcoR1* and *Xba1*. Products of ligation were transformed into XL 2 competent *E. coli* using thermoporation, and following selection and sequencing to confirm successful and accurate introduction of the correct sequence, plasmid was extracted from cultivated clones using the HiSpeed Plasmid Maxi Kit (QIAGEN). Human *MC3R* and *MC4R* clones used herein are as previously described (Yeo et al., 2003).

Activation by β-MSH and α-MSH of Melanocortin Receptors

COS-7 cells were transfected with *dMC3R*, *dMC4R*, *hMC4R*, or an empty vector and serum starved for 2 hr before stimulation with either α- or β-MSH in presence of IBMX. Ligands for use in receptor stimulation experiments were commercially available for α-MSH (peptide sequence identical in dogs and humans; Bachem) and human β-MSH (Bachem). Canine β-MSH was custom synthesized (Cambridge Research Biochemicals). The HitHunter cAMP XS+ Assay (DiscoverRx), a gain-of-signal competitive immunoassay based on enzyme fragment complementation technology, was used according to the manufacturer's recommendations to measure the downstream effect of receptor activation and cAMP generation.

Microsatellite Genotyping

Microsatellite markers 500 kb up- and downstream of the POMC mutation were identified from the University of California, Davis DOGSET database (Wong and Neff, 2009), and the suggested primer sequences were used to amplify and genotype three markers, 17_021E_CT, 17_022B_CA, and 17_022C_CA (DOGSET reference names). A two-stage amplification process was used: for the first stage, locus-specific forward primers appended with an M13 sequence tag at the 5′ end were used with standard reverse primers, and in the second stage, a FAM-labeled M13 primer was used with the same reverse primers to label PCR products before size separation against GeneScan 500 LIZ dye Size Standard (ThermoFisher Scientific) in the ABI 3700 Genetic Analyzer (Applied Biosystems) and analysis using GeneMapper software (ThermoFisher Scientific).

Clustal Alignment/Ensembl BLASTP for Sequence Similarity

We used the CLUSTAL Omega 2.1 multiple sequence alignment tool (Sievers et al., 2011) to align human and dog peptide and nucleotide sequences. Peptide sequence similarity was calculated using the NCBI BLASTP tool.

Accession Codes

Accession codes for nucleotide sequences used are as follows: dog *AGRP*, *MC4R*, and *POMC*, and human *POMC*, Ensembl: ENSCAFG00000020359, ENSCAFG00000000090, ENSCAFG00000004149, and ENSG00000115138, respectively. Accession code for dog *MC3R* is NCBI RefSeq: NC_006606.3. Peptide sequences for protein alignment for *Pomc* (mouse), *POMC* (dog), and *POMC* (human) were obtained from UniProt under accession codes UniProt: P01193, E2RQ39, and P01189, respectively.

Statistical Analysis

Descriptive statistics were generated to assess variance and normality of distribution prior to analysis. Fisher's exact test was used to test whether distribution was different between lean and obese groups for variants identified in candidate gene sequencing, and the chi-square test was used to test whether alleles were in Hardy-Weinberg equilibrium within populations. We investigated association of the deletion with body weight, adiposity, and food motivation score by linear regression models adjusted for age, sex, neuter status, and color (to account for their previously reported influence on body weight; Colliard et al., 2006; Pugh et al., 2015; Robertson, 2003; Sallander et al., 2010). Color was treated as a categorical variable (black, chocolate/liver, or yellow). Analyses were performed using Stata (StataCorp, 2015).

SUPPLEMENTAL INFORMATION

Supplemental Information includes one figure and four tables and can be found with this article online at http://dx.doi.org/10.1016/j.cmet.2016.04.012.

AUTHOR CONTRIBUTIONS

Conceptualization, E.R., S.O., and G.S.H.Y.; Methodology, E.R. and R.J.D.; Formal Analysis, E.R., R.J.D., S.P.S., and R.A.S.; Data Curation, E.R. and E.W.; Investigation, E.R., R.J.D., C.J.O., J.M.B., D.J.W., C.J.W., E.C., V.P.I., and E.W.; Writing – Original Draft, E.R., S.O., and G.S.H.Y.; Writing – Review & Editing, all authors; Visualization, E.R., R.J.D., and R.A.S.; Funding Acquisition, E.R. and S.O.; Resources, E.R., D.N.C., K.M.S., A.J.G., C.S.M., M.L.A., J.S., S.W., G.A., and K.L.-T.; Supervision, S.O. and G.S.H.Y.

ACKNOWLEDGMENTS

We are grateful to Rachel Moxon of Guide Dogs UK for collecting the assistance dog samples; Stephen J. Sharp of the MRC Epidemiology Unit for his statistical advice; Jens Häggström, Karin Hultin Jäderlund, and Berndt Klingeborn for the Swedish dog samples; Anne White for efforts to develop a canine beta MSH assay and adaptation of her original for Figure 1B; and the Dogslife Consortium for samples from British Labrador retrievers (supported by an Institute Core Strategic Grant from the BBSRC to the Roslin Institute). A full list of the investigators who contributed to the Dogslife project is available from www.dogslife.ac.uk/who-runs-dogslife. A.J.G.'s academic post at the University of Liverpool is financially supported by Royal Canin, and the same company has also provided research funding for this author. The work was primarily supported by the Wellcome Trust (Senior Investigator Award 095515/Z/11/Z and Strategic Award 100574/Z/12/Z), MRC (MRC Metabolic Diseases Unit, award 4050281695 and MRC_MC_UU_12012/5), and Dogs Trust. The authors would like to thank all the veterinary surgeons and nurses, owners, and dogs who contributed samples.

Received: January 19, 2016
Revised: March 16, 2016
Accepted: April 13, 2016
Published: May 3, 2016

REFERENCES

Appleyard, S.M., Hayward, M., Young, J.I., Butler, A.A., Cone, R.D., Rubinstein, M., and Low, M.J. (2003). A role for the endogenous opioid beta-endorphin in energy homeostasis. Endocrinology *144*, 1753–1760.

Challis, B.G., Pritchard, L.E., Creemers, J.W., Delplanque, J., Keogh, J.M., Luan, J., Wareham, N.J., Yeo, G.S., Bhattacharyya, S., Froguel, P., et al. (2002). A missense mutation disrupting a dibasic prohormone processing site in pro-opiomelanocortin (POMC) increases susceptibility to early-onset obesity through a novel molecular mechanism. Hum. Mol. Genet. *11*, 1997–2004.

Colliard, L., Ancel, J., Benet, J.J., Paragon, B.M., and Blanchard, G. (2006). Risk factors for obesity in dogs in France. J. Nutr. *136* (*Suppl 7*), 1951S–1954S.

Courcier, E.A., Thomson, R.M., Mellor, D.J., and Yam, P.S. (2010). An epidemiological study of environmental factors associated with canine obesity. J. Small Anim. Pract. *51*, 362–367.

Day, J.E.L., Kergoat, S., and Kotrschal, K. (2009). Do pets influence the quantity and choice of food offered to them by their owners: lessons from other animals and the pre-verbal human infant? CAB Reviews: Perspectives in Agriculture, Veterinary Science, Nutrition and Natural Resources. *4*, http://dx.doi.org/10.1079/PAVSNNR20094042.

Edney, A.T., and Smith, P.M. (1986). Study of obesity in dogs visiting veterinary practices in the United Kingdom. Vet. Rec. *118*, 391–396.

German, A.J. (2006). The growing problem of obesity in dogs and cats. J. Nutr. *136* (*Suppl 7*), 1940S–1946S.

Laflamme, D.P. (1997). Development and validation of a body condition score system for dogs. Canine Pract. *22*, 10–15.

Lawler, D.F., Larson, B.T., Ballam, J.M., Smith, G.K., Biery, D.N., Evans, R.H., Greeley, E.H., Segre, M., Stowe, H.D., and Kealy, R.D. (2008). Diet restriction and ageing in the dog: major observations over two decades. Br. J. Nutr. *99*, 793–805.

Lee, Y.S., Challis, B.G., Thompson, D.A., Yeo, G.S., Keogh, J.M., Madonna, M.E., Wraight, V., Sims, M., Vatin, V., Meyre, D., et al. (2006). A POMC variant implicates beta-melanocyte-stimulating hormone in the control of human energy balance. Cell Metab. *3*, 135–140.

Locke, A.E., Kahali, B., Berndt, S.I., Justice, A.E., Pers, T.H., Day, F.R., Powell, C., Vedantam, S., Buchkovich, M.L., Yang, J., et al.; LifeLines Cohort Study; ADIPOGen Consortium; AGEN-BMI Working Group; CARDIOGRAMplusC4D Consortium; CKDGen Consortium; GLGC; ICBP; MAGIC Investigators; MuTHER Consortium; MIGen Consortium; PAGE Consortium; ReproGen Consortium; GENIE Consortium; International Endogene Consortium (2015). Genetic studies of body mass index yield new insights for obesity biology. Nature *518*, 197–206.

Lund, E.M., Armstrong, P.J., Kirk, C.A., and Klausner, J.S. (2006). Prevalence and risk factors for obesity in adult dogs from private US veterinary practices. Intern J Appl Res Vet Med *4*, 177–186.

Mankowska, M., Stachowiak, M., Graczyk, A., Ciazynska, P., Gogulski, M., Nizanski, W., and Switonski, M. (2016). Sequence analysis of three canine adipokine genes revealed an association between TNF polymorphisms and obesity in Labrador dogs. Anim. Genet. *47*, 245–249 Published online December 22, 2015. http://dx.doi.org/10.1111/age.12390.

Mason, E. (1970). Obesity in pet dogs. Vet. Rec. *86*, 612–616.

Mawby, D.I., Bartges, J.W., d'Avignon, A., Laflamme, D.P., Moyers, T.D., and Cottrell, T. (2004). Comparison of various methods for estimating body fat in dogs. J. Am. Anim. Hosp. Assoc. *40*, 109–114.

McGreevy, P.D., Thomson, P.C., Pride, C., Fawcett, A., Grassi, T., and Jones, B. (2005). Prevalence of obesity in dogs examined by Australian veterinary practices and the risk factors involved. Vet. Rec. *156*, 695–702.

Mendez, I.A., Ostlund, S.B., Maidment, N.T., and Murphy, N.P. (2015). Involvement of endogenous enkephalins and β-endorphin in feeding and diet-induced obesity. Neuropsychopharmacology *40*, 2103–2112.

O Neill, D.G., Church, D.B., McGreevy, P.D., Thomson, P.C., and Brodbelt, D.C. (2014). Prevalence of disorders recorded in dogs attending primary-care veterinary practices in England. PLoS ONE *9*, e90501.

Pugh, C.A., Bronsvoort, B.M., Handel, I.G., Summers, K.M., and Clements, D.N. (2015). Dogslife: a cohort study of Labrador retrievers in the UK. Prev. Vet. Med. *122*, 426–435.

Raffan, E. (2013). The big problem: battling companion animal obesity. Vet. Rec. *173*, 287–291.

Raffan, E., Smith, S.P., O'Rahilly, S., and Wardle, J. (2015). Development, factor structure and application of the dog obesity risk and appetite (DORA) questionnaire. PeerJ *3*, e1278.

Robertson, I.D. (2003). The association of exercise, diet and other factors with owner-perceived obesity in privately owned dogs from metropolitan Perth, WA. Prev. Vet. Med. *58*, 75–83.

Sallander, M., Hagberg, M., Hedhammar, A., Rundgren, M., and Lindberg, J.E. (2010). Energy-intake and activity risk factors for owner-perceived obesity in a defined population of Swedish dogs. Prev. Vet. Med. *96*, 132–141.

Sievers, F., Wilm, A., Dineen, D., Gibson, T.J., Karplus, K., Li, W., Lopez, R., McWilliam, H., Remmert, M., Söding, J., et al. (2011). Fast, scalable generation of high-quality protein multiple sequence alignments using Clustal Omega. Mol. Syst. Biol. *7*, 539.

StataCorp. (2015). Stata statistical software: release 14 (StataCorp LP).

Ste Marie, L., Miura, G.I., Marsh, D.J., Yagaloff, K., and Palmiter, R.D. (2000). A metabolic defect promotes obesity in mice lacking melanocortin-4 receptors. Proc. Natl. Acad. Sci. USA *97*, 12339–12344.

Sutter, N.B., and Ostrander, E.A. (2004). Dog star rising: the canine genetic system. Nat. Rev. Genet. *5*, 900–910.

Tung, Y.C., Yeo, G.S., O'Rahilly, S., and Coll, A.P. (2014). Obesity and FTO: changing focus at a complex locus. Cell Metab. *20*, 710–718.

Vonholdt, B.M., Pollinger, J.P., Lohmueller, K.E., Han, E., Parker, H.G., Quignon, P., Degenhardt, J.D., Boyko, A.R., Earl, D.A., Auton, A., et al.

(2010). Genome-wide SNP and haplotype analyses reveal a rich history underlying dog domestication. Nature *464*, 898–902.

Wong, A.K., and Neff, M.W. (2009). DOGSET: pre-designed primer sets for fine-scale mapping and DNA sequence interrogation in the dog. Anim. Genet. *40*, 569–571.

Yeo, G.S., and Heisler, L.K. (2012). Unraveling the brain regulation of appetite: lessons from genetics. Nat. Neurosci. *15*, 1343–1349.

Yeo, G.S., Lank, E.J., Farooqi, I.S., Keogh, J., Challis, B.G., and O'Rahilly, S. (2003). Mutations in the human melanocortin-4 receptor gene associated with severe familial obesity disrupts receptor function through multiple molecular mechanisms. Hum. Mol. Genet. *12*, 561–574.

Zoran, D.L. (2010). Obesity in dogs and cats: a metabolic and endocrine disorder. Vet. Clin. North Am. Small Anim. Pract. *40*, 221–239.

Cell Metabolism, Volume *23*

Supplemental Information

A Deletion in the Canine *POMC* Gene Is Associated with Weight and Appetite in Obesity-Prone Labrador Retriever Dogs

Eleanor Raffan, Rowena J. Dennis, Conor J. O'Donovan, Julia M. Becker, Robert A. Scott, Stephen P. Smith, David J. Withers, Claire J. Wood, Elena Conci, Dylan N. Clements, Kim M. Summers, Alexander J. German, Cathryn S. Mellersh, Maja L. Arendt, Valentine P. Iyemere, Elaine Withers, Josefin Söder, Sara Wernersson, Göran Andersson, Kerstin Lindblad-Toh, Giles S.H. Yeo, and Stephen O'Rahilly

SUPPLEMENTAL INFORMATION – RAFFAN *ET AL*

SUPPLEMENTAL DATA ITEMS

Supplemental table 1 – Results of candidate gene sequencing. Related to Figure 1.

Variations (Var) from the canine reference genome (CanFam3.1) found on candidate gene sequencing. Number of dogs and their genotypes are shown. [a]V213F in MC4R has previously been shown not to affect receptor function[1]. [b]Deletion detailed in main paper. [c]rs22557623. [d]rs22557625. WT, wild type. *See Supplementalfigure 1.

Gene	Position in Ensembl CanFam3.1	Reference Allele	Mutant Allele	Consequence	Obese dogs			Lean dogs			p value of Fisher's exact test
					WT/WT	WT/Var	Var/Var	WT/WT	WT/Var	Var/Var	
MC4R	1:16132465	G	T	V213F	9	1	0	12	1	0	1.000[a]
	1:16132605	T	C	synonymous coding	0	0	9	0	0	13	Invariant
	1:16132817	G	T	S330I	9	0	0	12	1	0	1.000
	1:16132860	C	G	intronic	0	0	9	0	0	12	Invariant
	1:16133054	C	T	intronic	0	3	5	1	4	6	1.000
POMC	17:19431664	G	T	S239I	13	0	0	17	1	0	1.000
	**17:19431807**	**GCGCCGGCCCGGGA**	**-**	**P187fs**	**5**	**8**	**2**	**16**	**2**	**0**	**0.001[b]**
	17:19431813	C	G	synonymous coding	0	0	5	0	0	16	Invariant
	17:19431820	A	AG	P187PE*	0	0	15	0	0	18	Invariant
	17:19431850	C	G	R178G	0	0	12	0	0	17	Invariant
	17:19431861	A	G	K174R	0	0	12	0	0	17	Invariant
	17:19431983	C	G	synonymous coding	13	0	0	19	1	0	1.000
	17:19432208	G	A	synonymous coding	9	3	0	15	4	0	1.000
	17:19432226	C	A	synonymous coding	6	6	0	6	9	4	0.452
	17:19432391	GGGCGAGCTCCTG	-	intronic	7	4	0	10	7	0	1.000
	17:19432427	C	G	intronic	6	3	1	6	6	3	0.428[c]
	17:19432428	A	C	intronic	6	4	0	6	9	0	0.428[d]
	17:19434068	T	C	intronic	8	2	1	5	2	2	0.642

Supplemental table 2 – Breeds of dogs tested for both the *POMC* mutation and for naturally occurring variation in the *POMC* gene. Related to Figure 1.

	**Number of dogs tested for:**	
**Breed**	***POMC* mutation**	**Variation in *POMC* gene**
Beagle	17	2
Bearded collie	16	2
Bernese mountain dog	20	0
Border Collie	18	2
Boxer	17	2
Bull terrier	8	1
Cairn terrier	8	1
English springer spaniel	17	2
Finsk lapphund	8	1
Flat coat retriever	8	1
Giant schnauzer	16	2
Golden retriever	55	55
Great Dane	16	2
Hovawart	16	2
Irish wolfhound	16	2
Leonberger	8	1
Newfoundland	16	2
Polish lowland	8	1
Poodle	16	2
Rottweiler	20	0
Shar pei	16	2
Swedish elkhound	16	2
Welsh springer spaniel	16	2
Whippet	9	1
14 Other breeds: Crossbreed, cavalier King Charles spaniel, German shepherd, mastiff, shih tzu, Tibetan terrier, Border terrier, cocker spaniel, Dalmatian, dachshund, Doberman pinscher, pointer, Staffordshire bull terrier, West Highland white terrier	20	0

Supplemental Table 3 – Results of microsatellite genotyping in wild type and homozygous deletion Labrador retrievers and FCR. Related to figure 1.

Amplification of microsatellites 17_021D_CT (which lies 527 kb upstream of the deletion) and 17_022B_CA (66 kb downstream of the deletion) showed all FCR and Labrador retrievers homozygous for the POMC deletion were homozygous for the same microsatellite alleles, whereas wild type dogs carried various different alleles, evidence that the mutation is identical by descent in the two breeds. All map positions as per DOGSET (CanFam2)[2].

**DOGSET marker name**	**Physical/map position on Chromosome 17 bp/centimorgans**	**Alleles present (length of amplicon x no. of dogs with that allele)**			
		**Labrador retrievers**		**Flat coat retrievers**	
**17_021D_CT**	21885226/13.949	363/363 x 15<br>365/365 x 1	365/365 x 12	363/363 x 10<br>363/369 x 3<br>372/372 x 1	365/365 x 15
***POMC* deletion**	**22412852/13.949**	**Wild type**	**Homozygous Deletion**	**Wild type**	**Homozygous Deletion**
**17_022B_CA**	22478898/13.949	409/409 x 1<br>411/411 x 8<br>411/413 x 2<br>413/413 x 4	413/413 x 12	409/409 x 2<br>409/411 x 1<br>409/415 x 1<br>411/411 x 4<br>411/413 x 1<br>413/413 x 3	413/413 x 16
**17_022C_CA**	22558098/13.949	369/369 x 1<br>369/371 x 3<br>369/375 x 2<br>371/371 x 2<br>371/375 x 2<br>375/375 x 4	371/371 x 10<br>369/369 x 1<br>371/373 x 1	369/369 x 6<br>369/371 x 4<br>371/371 x 1<br>371/375 x 2	371/371 x 9<br>371/369 x 3<br>369/369 x 1

Supplemental Table 4 – Distribution of alleles in working and show strains of Labrador retrievers

There was no difference between the incidence of the mutation between dogs from ‘working’ and ‘show’ lines (Chi-squared test of wild type vs. carrier $p>0.3$). Related to figure 1.

	**Wild Type**	**Heterozygous**	**Homozygous Deletion**
**Working**	94	27	3
**Show**	21	9	1

Supplemental Figure 1 – Clustal Omega alignment of the CanFam3.1 reference sequence with sequence in Labrador retrievers and other breeds for exons 3-4 of POMC. Related to Figure 1. Alignment annotated to show variation from the reference sequence identified in Labrador retrievers and other breeds (see Supplementaltable 1). Underlined bases denote position of frameshift deletion. (a) 17: 19431861, A>G in all dogs sequenced with consequence p.K174R, which changes the distal di-basic cleavage site for ACTH to 'KR' from 'KK' which is the same as the human reference sequence. (b) 17: 19431850 C>G in all dogs sequenced, consequence p.R178G which is identical to the human reference sequence. (c) 17:19431821 C>CG. This single base pair insertion at the start of what is annotated as a two base pair intron (highlighted blue) has the consequence p.P187>PE which results in closer homology to the human reference sequence. (d) 17:19431813 C>G in all dogs sequenced, synonymous coding. (e) 17:19431777 C>T, novel synonymous coding SNP. (f) 17:19431764 G>T, p.E206D, not found in Labrador retrievers or FCR.

```
CLUSTAL O(1.2.1) multiple sequence alignment


POMC_reference_CanFam3.1        GCGTGCATCCGGGCCTGCAAGCCCGACCTCTCCGCCGAGACGCCCGTGCTCCCCGGCAAC
All_breed_consensus             GCGTGCATCCGGGCCTGCAAGCCCGACCTCTCCGCCGAGACGCCCGTGCTCCCCGGCAAC
                                ************************************************************

POMC_reference_CanFam3.1        GGCGACGAGCAGCCGCTGGCTGAGAACCCCCGGAAGTACGTCATGGGCCACTTCCGCTGG
All_breed_consensus             GGCGACGAGCAGCCGCTGGCTGAGAACCCCCGGAAGTACGTCATGGGCCACTTCCGCTGG
                                ************************************************************

POMC_reference_CanFam3.1        GACCGGTTTGGCCGCCGCAATGGCAGCGCGGGCCAGAAGCGCGAGGAAGAAGAGGTGGCG
All_breed_consensus             GACCGGTTTGGCCGCCGCAATGGCAGCGCGGGCCAGAAGCGCGAGGAAGAAGAGGTGGCG
                                ************************************************************

POMC_reference_CanFam3.1        GCGGGCGGAGGCCGCGCCCCGCTGCCCGCGGGCGGCCCGGGGCCCCGCGGCGACGGTGGC
All_breed_consensus             GCGGGCGGAGGCCGCGCCCCGCTGCCCGCGGGCGGCCCGGGGCCCCGCGGCGACGGTGGC
                                ************************************************************

POMC_reference_CanFam3.1        GAGCTCGGCCTGCAAGAGGGCAAGCGCTCCTACTCCATGGAGCACTTCCGCTGGGGCAAG
All_breed_consensus             GAGCTCGGCCTGCAAGAGGGCAAGCGCTCCTACTCCATGGAGCACTTCCGCTGGGGCAAG
                                ************************************************************

POMC_reference_CanFam3.1        CCGGTGGGCAAGAAGCGGCGCCCGGTGAAGGTGTACCCCAACGGCGCTGAGGACGAGTCG
All_breed_consensus             CCGGTGGGCAAGAAGCGGCGCCCGGTGAAGGTGTACCCCAACGGCGCTGAGGACGAGTCG
                                ************************************************************

POMC_reference_CanFam3.1        GCCGAGGCCTTCCCCGTCGAGTTCAAGAAGGAGCTGGCCCGGCAGCGGCTGGAGCCGGCG
All_breed_consensus             GCCGAGGCCTTCCCCGTCGAGTTCAAGAGGGAGCTGGCCGGGCAGCGGCTGGAGCCGGCG
                                **************************** ********** ********************
                                                           (a)        (b)
POMC_reference_CanFam3.1        CTCGGCCCC-AGGGCCCCGCCGCGGGCGTGGCGGCGCTGGCCGACCTGGAGTACGGCCTG
All_breed_consensus             CTCGGCCCCGAGGGCCCGGCCGCGGGCGTGGCGGCGCTGGCCGACCTGGAGTAYGGCCTG
                                ********* ******* *********************************** ******
                                        (c)     (d)                                (e)
POMC_reference_CanFam3.1        GTGGCGGAGGCCGGGGCGGCCGAGAAGAAGGACGACGGGCCCTACAAGATGGAGCACTTC
All_breed_consensus             GTGGCGKAGGCCGGGGCGGCCGAGAAGAAGGACGACGGGCCCTACAAGATGGAGCACTTC
                                ****** *****************************************************
                                     (f)
POMC_reference_CanFam3.1        CGCTGGGGCAGCCCGCCCAAGGACAAGCGCTACGGCGGCTTCATGAGCTCGGAGAGGAGC
All_breed_consensus             CGCTGGGGCAGCCCGCCCAAGGACAAGCGCTACGGCGGCTTCATGAGCTCGGAGAGGAGC
                                ************************************************************

POMC_reference_CanFam3.1        CAGACGCCCCTGGTGACGCTGTTCAAAAACGCCATCATCAAGAACGCCCACAAGAAGGGC
All_breed_consensus             CAGACGCCCCTGGTGACGCTGTTCAAAAACGCCATCATCAAGAACGCCCACAAGAAGGGC
                                ************************************************************

POMC_reference_CanFam3.1        CAGTGA
All_breed_consensus             CAGTGA
                                ******
```

Supplemental References

1 Yan, J. & Tao, Y. X. Pharmacological characterization of canine melancortin-4 receptor and its natural variant V213F. *Domestic animal endocrinology* **41**, 91-97, doi:10.1016/j.domaniend.2011.05.002 (2011).

2 Wong, A. K. & Neff, M. W. DOGSET: pre-designed primer sets for fine-scale mapping and DNA sequence interrogation in the dog. *Animal genetics* **40**, 569-571, doi:10.1111/j.1365-2052.2009.01875.x (2009).
